# Comparative Analysis of *PvPAP* Gene Family and Their Functions in Response to Phosphorus Deficiency in Common Bean

**DOI:** 10.1371/journal.pone.0038106

**Published:** 2012-05-25

**Authors:** Cuiyue Liang, Lili Sun, Zhufang Yao, Hong Liao, Jiang Tian

**Affiliations:** 1 State Key Laboratory for Conservation and Utilization of Subtropical Agro-bioresources, Root Biology Center, South China Agricultural University, Guangzhou, People’s Republic of China; 2 Robert Holley Center for Agriculture and Health, United States Department of Agriculture, Agricultural Research Service, Cornell University, Ithaca, New York, United States of America; 3 Institute of Tropical Crop Genetic Resources, Chinese Academy of Tropical Agriculture Sciences, Danzhou, China; United States Department of Agriculture, Agricultural Research Service, United States of America

## Abstract

**Background:**

Purple acid phosphatases (PAPs) play a vital role in adaptive strategies of plants to phosphorus (P) deficiency. However, their functions in relation to P efficiency are fragmentary in common bean.

**Principal Findings:**

Five *PvPAP*s were isolated and sequenced in common bean. Phylogenetic analysis showed that PvPAPs could be classified into two groups, including a small group with low molecular mass, and a large group with high molecular mass. Among them, PvPAP3, PvPAP4 and PvPAP5 belong to the small group, while the other two belong to the large group. Transient expression of *35S:PvPAPs*-*GFP* on onion epidermal cells verified the variations of subcellular localization among PvPAPs, suggesting functional diversities of PvPAPs in common bean. Quantitative PCR results showed that most *PvPAPs* were up-regulated by phosphate (Pi) starvation. Among them, the expression of the small group *PvPAPs* responded more to Pi starvation, especially in the roots of G19833, the P-efficient genotype. However, only overexpressing *PvPAP1* and *PvPAP3* could result in significantly increased utilization of extracellular dNTPs in the transgenic bean hairy roots. Furthermore, overexpressing *PvPAP3* in Arabidopsis enhanced both plant growth and total P content when dNTPs were supplied as the sole external P source.

**Conclusions:**

The results suggest that PvPAPs in bean varied in protein structure, response to P deficiency and subcellular localization. Among them, both PvPAP1 and PvPAP3 might function as utilization of extracellular dNTPs.

## Introduction

Phosphorus (P), a critical macronutrient for plant growth, is directly or indirectly involved in many metabolic processes. Since terrestrial plants prefer to acquire P in its inorganic form (phosphate, Pi), which is easily fixed by soils, low P availability is one of the major limiting factors for plant growth in soils, especially in tropical and subtropical areas [1], [2]. In order to adapt to unfavorable conditions, plants have developed a set of strategies to improve P acquisition and utilization. These strategies include modification of root morphology and architecture to acquire more available P in soils [3], [4], increase of acid phosphatase (APase) activities [5]–[7] and organic acid exudation [8] to release Pi from the unavailable forms (e.g., Fe/Al-P complexes), as well as enhanced expression of a diverse array of genes [1], [2]. Since 50% to 80% of the total P in soils exists as organic P with the major form as monoester P, APases are generally assumed to play an important role in P acquisition [9]–[11].

Plant APases (EC 3.1.3.2) hydrolyze Pi from orthophosphoric monoesters with acidic pH optima. Among them, purple acid phosphatase (PAP) is a special group of APases characterized by the purple color of purified protein in water solution, metallo-phosphatase activity, and a lack of tartrate inhibition [12]–[15]. Comparative analysis of multiple PAP sequences from organisms resulted in identification of seven invariant residues in five conserved motifs (**D**XG/G**D**XX**Y**/G**N**H(D/E)/VXX**H**/G**H**X**H**; bold letters represent metal-ligating residues), which are required for metal coordination to form a dimetal nuclear center [13], [14]. Based on molecular mass and protein structure, PAPs can be further divided into two groups, small PAPs with a molecular mass of about 35 kD, and large PAPs that are mostly homodimeric proteins with a subunit molecular mass of about 55 kD [12]–[15].

Through biochemical analysis of purified native PAPs, the biochemical properties of some PAPs have been characterized in tomato (*Lycopersicon esculentum*), soybean (*Glycine max)*, common bean (*Phaseolus vulgaris*), Arabidopsis (*Arabidopsis thaliana*) and lupin (*Lupinus luteus*) [5], [6], [16]–[20]. All of these PAPs showed activities against Pi esters. Furthermore, some PAPs have been shown to have phytase activity, such as GmPhy in soybean and AtPAP15 in Arabidopsis [18]–[20]. Unfortunately, the optimum non-synthetic substrates have not been documented for most plant PAPs. Therefore, it is difficult to infer the functions of the most PAPs in plants.

With the availability of genomic resources, many *PAP* genes have been identified in plants, such as Arabidopsis and soybean [5], [14], [21]. For example, 29 potential *PAP* members have been identified in Arabidopsis [14]. Comparative analysis of expression patterns showed that the transcripts of at least three *AtPAPs* (*AtPAP11, AtPAP12 and AtPAP17*) were up-regulated by P deficiency [5], [14]. However, systematic analysis of *PAP* expression patterns as related to P availability is very limited in other crop plants.

Common bean is a major food leguminous crop, mostly grown on P deficient soils [3]. Two common bean genotypes, G19833 (P-efficient) and DOR364 (P-inefficient), as well as their recombinant inbred lines (RILs), have been used to investigate the mechanisms of bean adaptation to P deficiency. Adaptive strategies might include formation of shallower root systems, more exudation of organic acids and protons, and development of more cortical aerenchyma in roots [3], [22], [23]. Recently, genes involved in the response network of common bean to P deficiency have been identified [24]–[26], and some genes or ESTs encoding PAPs have been deposited in public databases. However, *PAP* expression patterns as related to Pi starvation remain unknown, except that expression levels of one EST (FD795350) and one gene *PvPAP3* (AC025293) have been found to be up-regulated by Pi starvation [25], [27]. In the present study, a search of genes or ESTs encoding PAPs was conducted in public databases, which resulted in retrieval of four *PvPAPs*. Systematic analysis of expression patterns and subcellular localization of the *PvPAPs* were further investigated. Subsequently, functions of PvPAPs involved in extracellular dNTP utilization were assayed.

## Results

### Isolation of *PvPAP5*, Phylogenetic Analysis and Alignment of PvPAPs

A search conducted at the NCBI website yielded four reported PAPs in common bean, including PvPAP1 (BAD05166), PvPAP2 (CAA04644), PvPAP3 (AC025293) and PvPAP4 (AAF60317).

Using the sequence of *PvPAP4* as a query sequence, two ESTs, FD786924 and TC7351, were identified from the DFCI *Phaseolus vulgaris* Gene Index (http://compbio.dfci.harvard.edu/tgi/). Based on the sequence of FD786924, the full length cDNA of *PvPAP5* was cloned. *PvPAP5* contains a 980-bp open reading frame encoding a polypeptide of 326 amino acid residues with a theoretical isoelectric point (pI) and a molecular mass of 5.5 and 36.9 kD, respectively. Furthermore, encoding regions for *PvPAP3*, *PvPAP4* and *PvPAP5* exhibited no difference between G19833 and DOR364 (data no shown).

**Figure 1:**
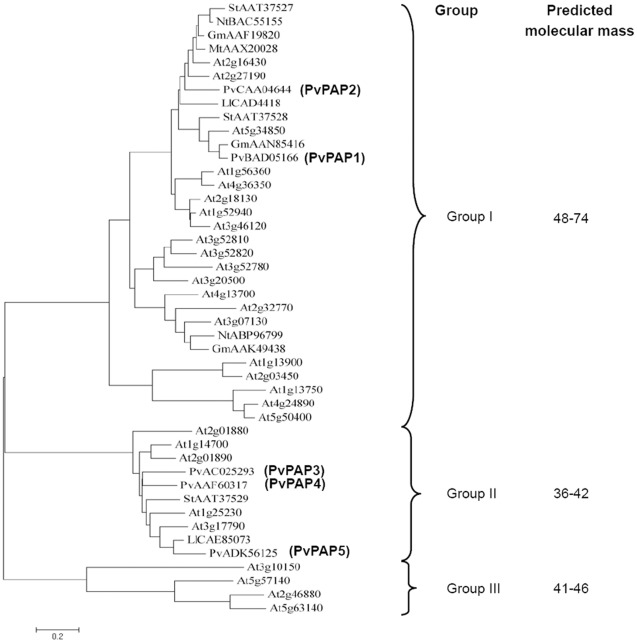
Phylogenetic analysis of PAP proteins in plants. For Arabidopsis, the AGI symbol is presented, while for other species, the first two letters of each protein label represent the abbreviated species name, which is followed by the GenBank accession number. Gm: *Glycine max*; LI: *Lupinus luteus*; Mt: *Medicago truncatula*; Nt: *Nicotiana tabacum*; Pv: *Phaseolus vulgaris*; St: *Solanum tuberosum*. The ranges of protein molecular mass are also listed.

A phylogenetic tree was generated through analysis of the PvPAPs in common bean and some other PAPs in plants, including Arabidopsis (*Arabidopsis thaliana*), potato (*Solanum tuberosom*), soybean (*Glycine max)*, lupin (*Lupinus luteus*), tobacco (*Nicotiana tabacum*) and *Medicago truncatula*. Phylogenetic analysis showed that there were three distinct PAP groups (Figure 1). The molecular mass of proteins in the group I was the highest, followed by group III and II. PvPAP1 and PvPAP2 belonged to the group I, separately exhibiting high homology with GmPAP3 (AAN85416) and AtPAP12 (At2g27190), while PvPAP3, PvPAP4 and PvPAP5 were included in the group II together with AtPAP17 (At3g17790), and could be considered as small PAPs (Figure 1).

The amino acid alignments of the two large PvPAPs and three small PvPAPs are illustrated in Figure S1. Like other reported PAPs in plants, seven invariant residues (**D**XG/G**D**XX**Y**/G**N**H/VXX**H**/G**H**X**H**) (the highlighted bold letters) were found in the five conserved motifs of PvPAPs, which are generally believed to be required for metal coordination.

### Subcellular Localization of PvPAPs in Onion Epidermal Cells

**Figure 2:**
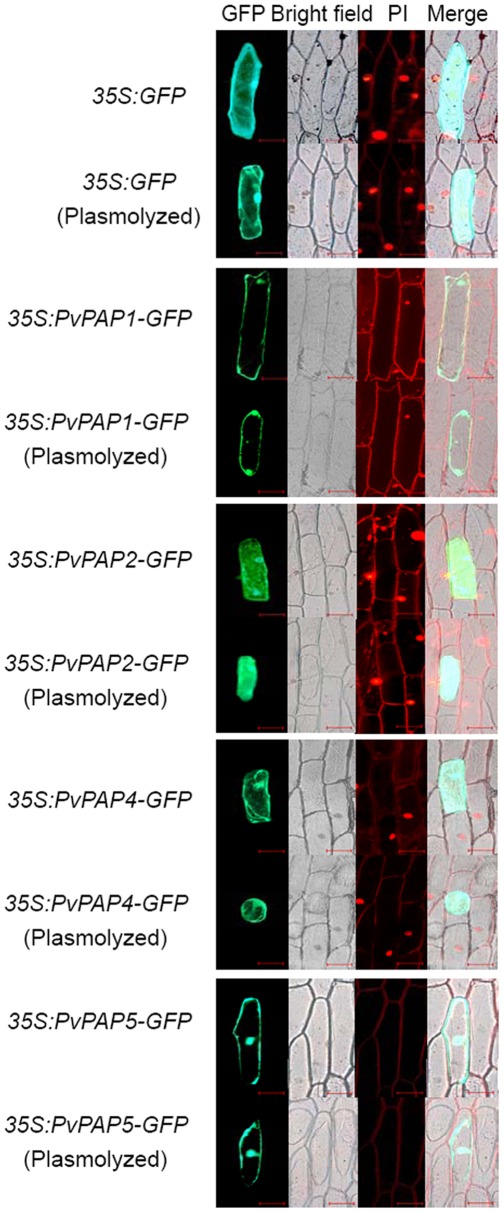
Subcellular localization of PvPAPs fused to GFP protein in onion epidermal cells. The top two rows show the empty vector control before and after plasmolyzing, followed by *PvPAP1-GFP*, *PvPAP2-GFP*, *PvPAP4-GFP*, and *PvPAP5-GFP* constructs before and after plasmolyzing. Cells were observed by green GFP fluorescence of the GFP and the PvPAP-GFP proteins and red propidium iodide (PI) fluorescence of the cell wall. Bars = 100 µm.

To determine subcellular localization of the PvPAPs, the *PvPAPs* genes were combined with a *GFP* reporter gene and transiently expressed in onion epidermal cells. Variations in the subcellular localization of the PvPAPs were observed through detection of GFP signals in the transformed onion epidermal cells (Figure 2). The signal of GFP in the empty vector control was detected throughout intracellular areas. However, GFP fluorescence of the large group PvPAPs-GFP was separately detected in plasma membrane and nucleus for PvPAP1-GFP, throughout the protoplasm for PvPAP2-GFP. Since subcellular localization of PvPAP3 to the plasma membrane and apoplast has previously been demonstrated in onion epidermal cells [27], subcellular localization of the other two small PvPAPs, including PvPAP4 and PvPAP5 was further analyzed in the study. Similar to the subcellular localization of the PvPAP2, PvPAP4 was localized throughout the intracellular areas, but PvPAP5 was localized in plasma membrane and nucleus. Variations in subcellular localization of PvPAPs strongly suggest the complex functions in bean.

### Internal APase Activities as Affected by P Deficiency

**Figure 3:**
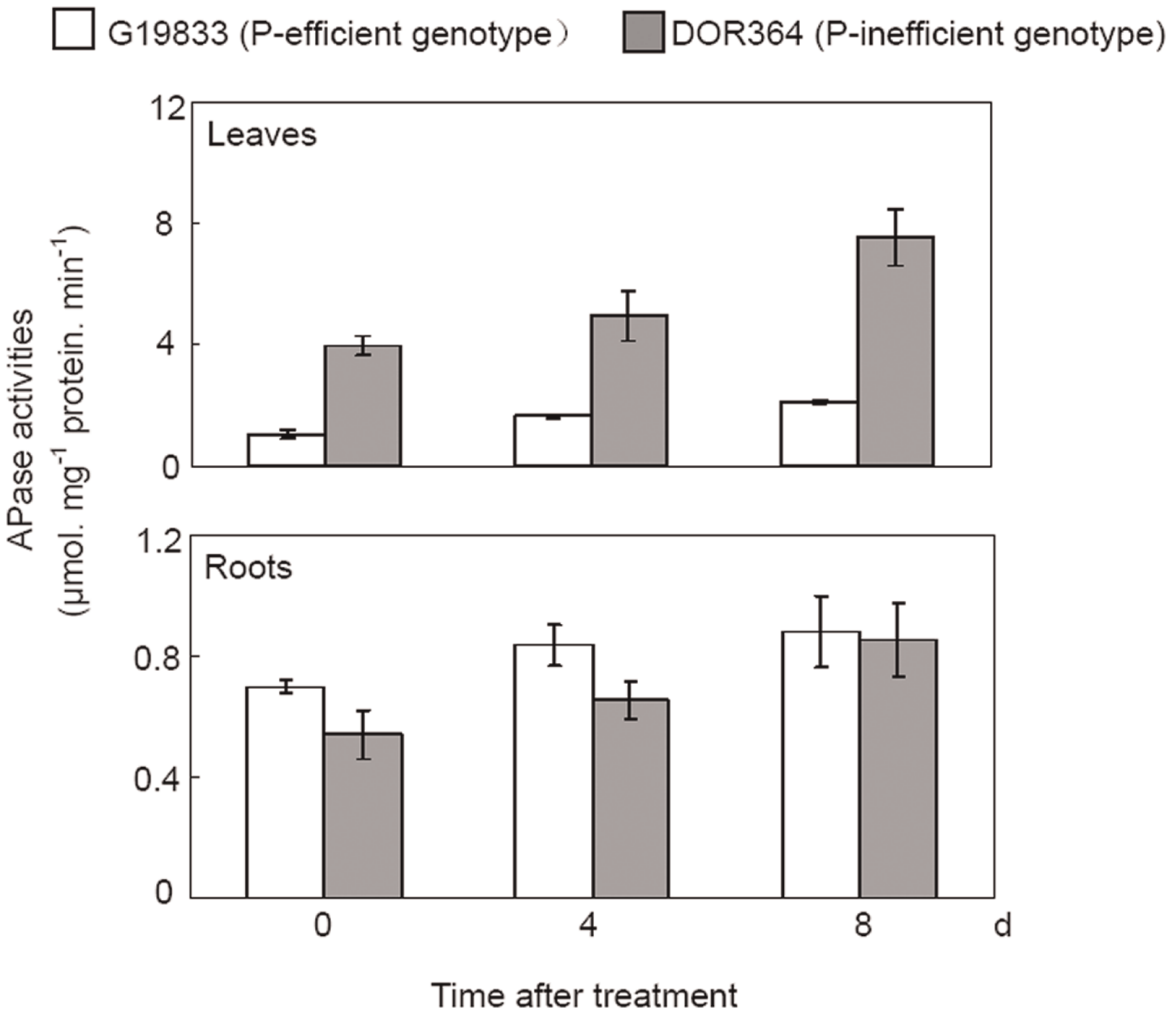
Dynamics of APase activities in leaves and roots in common bean during Pi starvation. *F* value of ANOVA: Leaves, 81.61 for genotype (*p*<0.05); 9.98 for P treatment (*p*<0.05); Roots, 3.12 for genotype (NS); 4.26 for P treatment (*p*<0.05). NS means the difference is not significant at 0.05 level. Each bar shows the mean of four replicates with standard error.

Throughout the duration of P deficiency, inhibited growth of both G19833 and DOR364 was observed, especially at 8 d after Pi starvation (data not shown). However, internal APase activities in leaves and roots of both genotypes were increased (Figure 3). Increases of internal APase activities were generally greater in leaves than in roots after 8 d of Pi starvation, as indicated by 102% and 89% increase in the leaves of G19833 and DOR364, respectively. Furthermore, internal APase activities in the leaves of DOR364 (P-inefficient genotype) were significantly higher than those of G19833 (P-efficient genotype). But no such genotypic differences existed in roots (Figure 3).

### Differential Responses of *PvPAPs* to Pi Starvation

**Figure 4:**
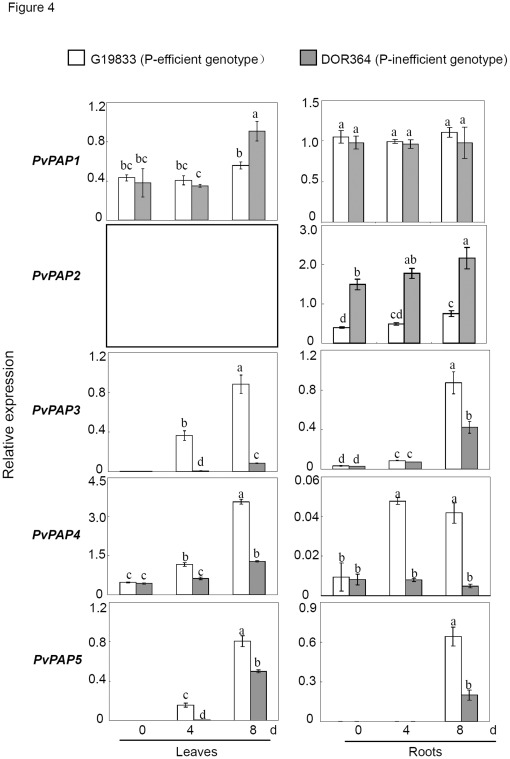
Transcripts of *PvPAPs* in leaves and roots during Pi starvation in common bean. PvTC3216 (*EF-1α*) was used as an internal control. Expression of the *PvPAP*s is shown as relative to the *EF-1α* reference using arbitrary units. Each bar shows the mean of four replicates with standard error. Different letters mean significant difference.

Temporal and tissue-specific expression patterns of *PvPAPs* in response to Pi starvation were comparatively analyzed through the qPCR. The results showed that transcripts of the *PvPAPs* varied greatly during Pi starvation (Figure 4). In general, only *PvPAP1* was constitutively expressed in roots, while the other *PvPAPs* were increased by Pi starvation in at least one genotypes or one tissue (Figure 4).

In the large group *PAP*, *PvPAP1* was constitutively expressed in roots and only enhanced at 8 d of Pi starvation in leaves of DOR364. *PvPAP2* was specifically expressed in roots, and its expression was enhanced with duration of Pi starvation. Furthermore, expression levels of *PvPAP1* and *PvPAP2* in G19833, the P-efficient genotype, were close or lower than those in DOR364, the P-inefficient genotype (Figure 4).

Interestingly, the transcripts of three small *PvPAPs* were dramatically increased during Pi starvation in both genotypes, except that constitutive expression pattern of *PvPAP4* was observed in the roots of DOR364 (Figure 4). Furthermore, the transcripts of the small *PvPAPs* in G19833 were significantly more than those in DOR364, especially at 8 d after Pi starvation. For example, expression levels of *PvPAP3* in the leaves and roots of G19833 were 10 and 2 times higher than those in DOR364 at 8 d of P deficiency, respectively.

### Utilization of Extracellular dNTPs in tTransgenic Bean Hairy rRoots

**Figure 5:**
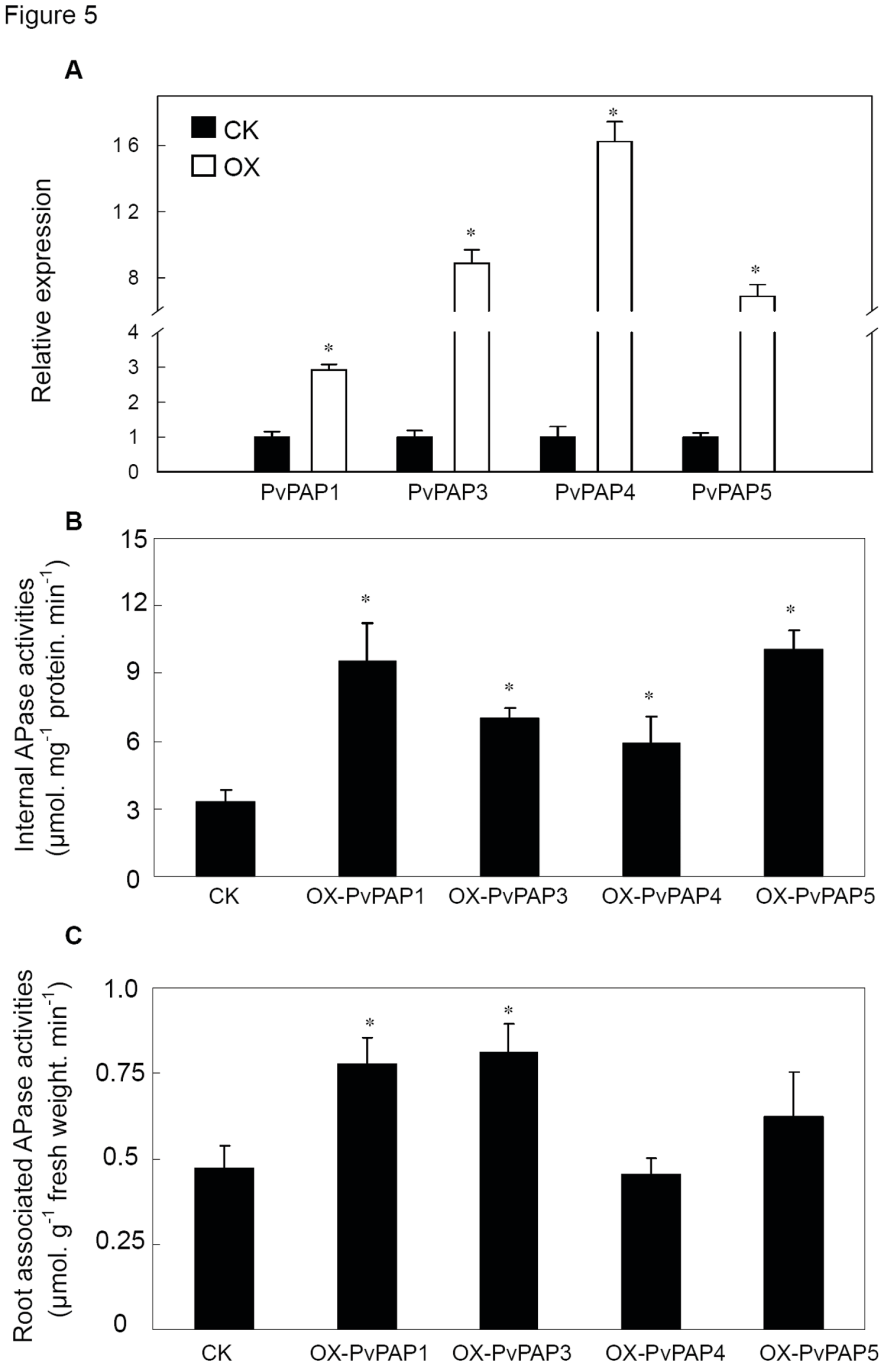
Transcripts and APase activities in the transgenic hairy roots. A) Transcripts of *PvPAPs* in the transgenic hairy roots. B) Internal APase activities; C) Root associated APase activities; Expression levels of *PvPAP1*, *PvPAP3*, *PvPAP4* and *PvPAP5* were normalized by their expression levels in the transgenic lines transformed with the empty vector, respectively. CK represents transgenic line transformed with the empty vector; OX-PvPAP1,3–5 means transgenic hairy roots with overexpressing *PvPAP1*, *PvPAP3*, *PvPAP4* and *PvPAP5*, respectively. Each bar is the mean of four biological replicates with standard error. Star symbols represent significant difference between the CK line and OX-PvPAP lines through student’s *t* test (*p*<0.05).

In order to further investigate the functions of *PvPAPs*, the transgenic bean hairy roots with overexpressing *PvPAPs* were successfully generated except for *PvPAP2* because overexpressing *PvPAP2* seemed toxic to hairy root growth. Results showed that increased transcripts and internal APase activities were observed in the transgenic hairy roots with overexpressing *PvPAP1*, *PvPAP3*, *PvPAP4* and *PvPAP5*, respectively (Figure 5A and B). However, significantly increased root associated APase activities were only observed in the transgenic hairy roots with *PvPAP1* and *PvPAP3* overexpression (Figure 5C). Root associated APase activities in OX-PvPAP1 and OX-PvPAP3 were 65% and 72% higher than those in CK, respectively (Figure 5C).

**Figure 6:**
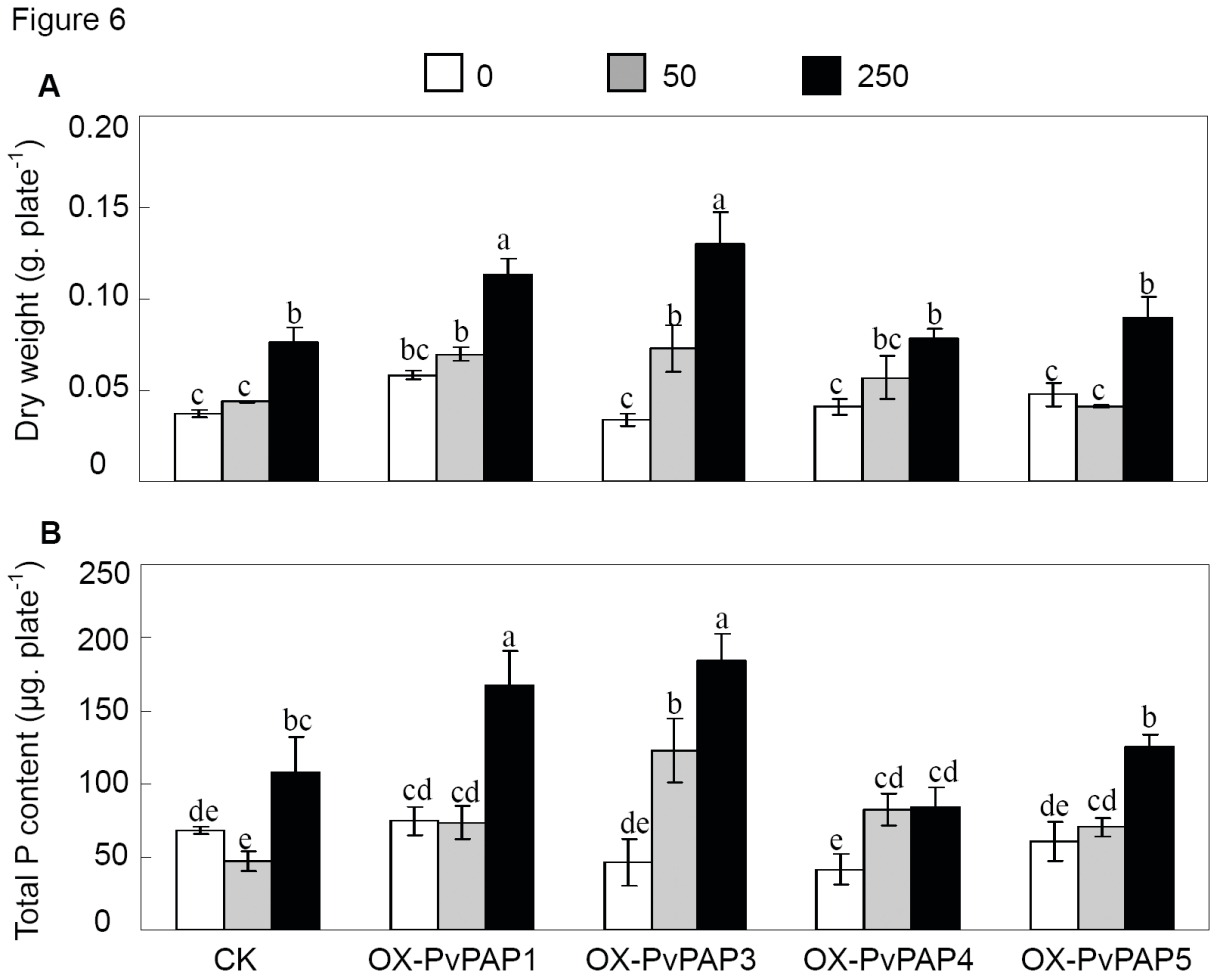
Dry weight and total P content of hairy roots at different levels of dNTPs. A) Dry weight; B) Total P content. CK represents bean hairy roots transformed with the empty vector; OX-PvPAP1,3–5 means transgenic bean hairy roots with overexpressing *PvPAP1*, *PvPAP3*, *PvPAP4* and *PvPAP5*, respectively. Hairy roots were grown in MS medium without KH_2_PO4 application, but with 0, 50 or 250 µM dNTP supplication for 7 d. Dry weight and total P content were separately measured. Each bar shows the mean of five replicates with standard error. Different letters mean significant difference (*p*<0.05).

Subsequently, functions of the PvPAPs as related to extracellular dNTP utilization were investigated in transgenic bean hairy roots. Overexpressing *PvPAP1* and *PvPAP3* resulted in enhanced dNTP utilization, as reflected by the highest dry weight and total P content in the transgenic hairy roots with 250 µM dNTP addition (Figure 6). Without dNTP supply, similar dry weight and total P content were observed in all the bean hairy roots except that *OX-PvPAP4* hairy roots had slightly lower total P content (Figure 6B). Although increased dry weight and total P content were observed for all the hairy roots with 250 µM dNTP application, dry weight and total P content in the *OX-PvPAP1* and *OX-PvPAP3* hairy roots were significantly increased, and higher than those in the CK hairy roots (Figure 6).

### *PvPAP3* Functions in Extracellular dNTP Utilization in Transgenic Arabidopsis Plants

**Figure 7:**
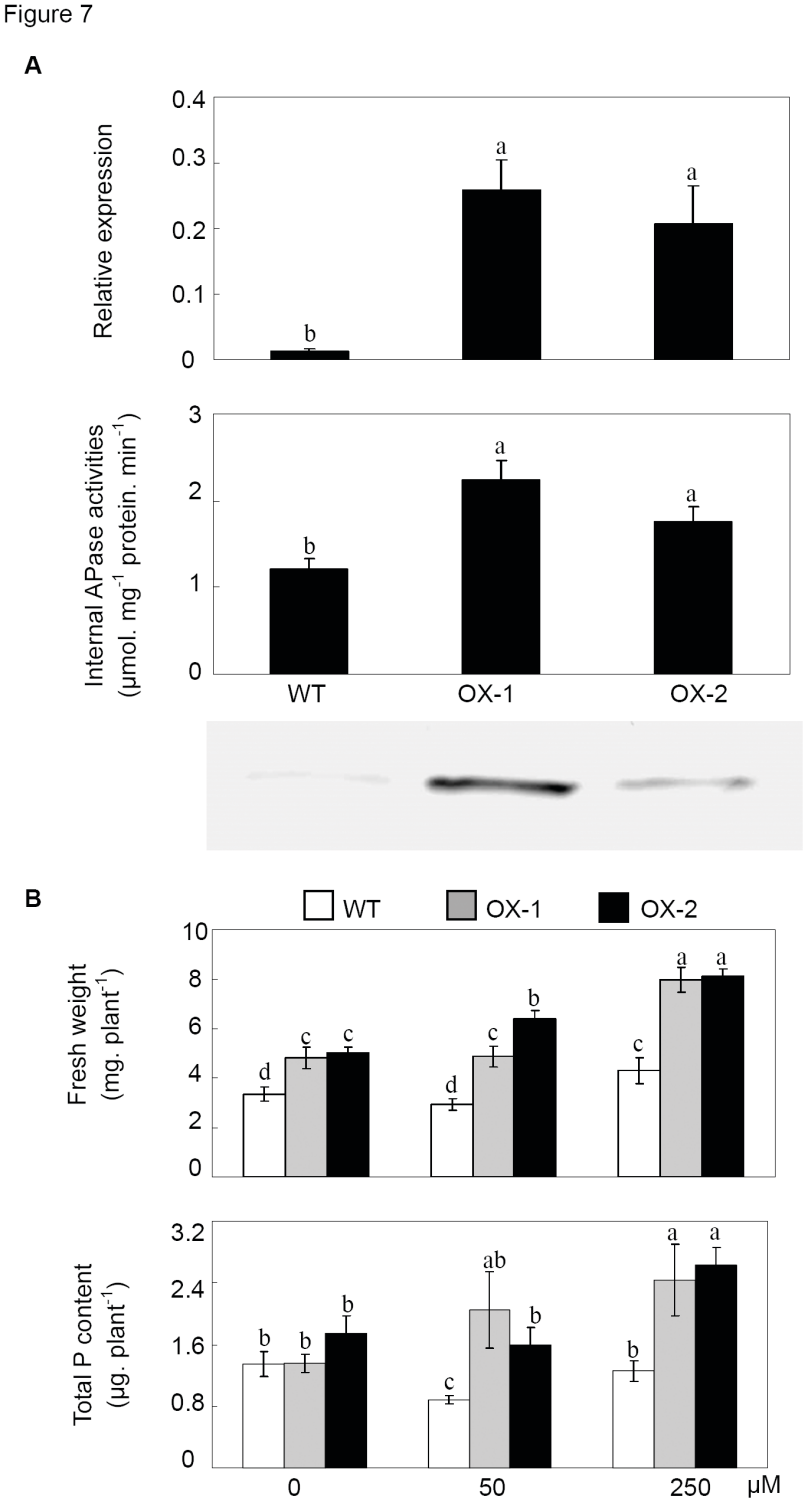
Utilization of dNTPs in transgenic Arabidopsis with *PvPAP3* overexpression. A) qPCR analysis, APase activities and western blot of *PvPAP3* transcripts in the transgenic plants. Each bar shows the mean of four replicates with standard error; B) Fresh weight and total P content of plants supplied with different dNTPs. WT represents wild-type plants; Two over expressing *PvPAP3* transgenic lines are separately shown as OX-1 and OX-2. Plants were grown in MS medium without KH_2_PO_4_ application, but with 0, 50 or 250 µM dNTP supplication. Each bar shows the mean of four replicates with standard error. Different letters mean significant difference (*p*<0.05).

Functions of *PvPAP3* as related to extracellular dNTP utilization were further investigated in two *PvPAP3*-overexpression Arabidopsis transgenic lines. These two transgenic lines overexpressing *PvPAP3* were generated, and verified through qPCR analysis and western blot analysis using a PvPAP3 antibody (Figure 7A). Results showed that accumulation of PvPAP3 led to increase of internal APase activities in the two transgenic lines by 85% and 45%, respectively (Figure 7A). Subsequently, wild-type and *PvPAP3*-overexpressing plants were grown in the media supplied with 0, 50 and 250 µM dNTPs, respectively. Enhanced utilization of external dNTPs in both transgenic lines was reflected by significant increase of fresh weight and total P content in *PvPAP3*-overexpressing plants when dNTPs were supplied as the sole external P source (Figure 7B).

Fresh weight and total P content of two transgenic lines were significantly increased with the increase of external dNTPs in the media (Figure 7B). When external dNTPs were 250 µM, total P content of the two transgenic lines were increased by 80% and 50%, respectively. However, fresh weight and total P content in the wild-type plants were slightly increased and lower than those of the transgenic plants with the increase of dNTPs in the growth media (Figure 7B). These results indicate augmentation of P acquisition from extracellular dNTPs by overexpressing *PvPAP3* in Arabidopsis plants.

## Discussion

Purple APases are believed to be important for P metabolism, and have been widely found among different organisms, including plants, animals and fungi [28]. Recently, accumulating results have supported the involvement of some PAPs in improving P efficiency in plants. For example, in Arabidopsis, overexpression of *MtPAP1* (AAX20028) resulted in increase of the secreted APase activities, which subsequently enhanced utilization of organic P in the media [29]. Overexpressing *AtPAP15* (At3g07130) has also been documented to improve P efficiency in soybean [7]. However, systematic analysis of their transcripts responsive to P availability and their specific functions in extracellular dNTP utilization has not been well investigated in crops. In the present study, comparative analysis of *PvPAPs* was conducted in common bean from primary structure, expression patterns as affected by Pi starvation, subcellular localization to functions. Although seven invariant residues required for metal coordination were observed for all the five PvPAPs, diversities of the primary structure of PvPAPs were observed (Figure 1 and S1), indicating they might have different functions in common bean, which was further demonstrated by their various responses to P deficiency and different subcellular localization (Figure 2 and 4), and utilization of extracellular dNTPs in the transgenic bean hairy roots (Figure 6).

Enhanced expression levels of *PAPs* by Pi starvation have been documented in plants, such as Arabidopsis [5], [14], common bean [27], potato [30]. However, most Pi-starvation responsive PAPs belonged to large PAPs in plants, such as three AtPAPs (At2g27190, At2g18130 and At5g34850) in Arabidopsis, and two StPAPs (StAAT37528, StAAT37527) in potato (Figure 1) [14], [30]–[31]. Only two small *PAPs* have been reported to be up-regulated by Pi starvation, including *AtPAP17* (At3g17790) and *PvPAP3* [5], [27]. In the study, only *PvPAP1* was constitutively expressed in roots, and the other *PvPAPs* were increased by Pi starvation in at least one genotypes or one tissue (Figure 4). Furthermore, transcripts of all the three small *PvPAPs* exhibiting high homology with small *AtPAP17* (At3g17790) were significantly increased by P deficiency in common bean (Figure 4). These results suggest that some *PvPAP*s may function under most or all conditions, while others are regulated to function in response to Pi-starvation.

Despite the accumulated knowledge on identification and biochemical characterization of plant PAPs, information on subcellular localization of PAPs in plants is limited. Recently, localization of several plant PAPs has been documented, including GmPAP3 in mitochondria [32], AtPAP26 in vacuole [33], PvPAP3 in plasma membrane and apoplast [27], NtPAP12 and AtPAP10 in cell wall [34], [35], AtPAP12 and AtPAP26 in seretome [31], [33]. Unlike all of these reports of the subcellular localization of plant PAPs, the PvPAPs in the study were either separately localized in the plasma membrane and nucleus (PvPAP1 and PvPAP5) or intracellular areas (PvPAP2 and PvPAP4) (Figure 2), indicating various functions among the PvPAPs, and these PvPAPs in bean might function in reactions or pathways that are different from PAPs previously studied in plants.

Since it has been demonstrated that microbial P could reach 30–40% of total P in soils, in which 35–65% of P-containing compounds were nucleic acids [36], it might be possible that utilization of the hydrolyzed Pi from extracellular dNTPs could be one of the strategies for plant adaptation to environments that are low in P availability but rich in organic P matter. In order to verify whether PvPAPs are involved in effective extracellular dNTP utilization, transgenic bean hairy roots with overexpressing *PvPAPs* were generated and analyzed. Although internal APase activities in bean hairy roots were greatly increased through overexpressing *PvPAPs*, significantly increased root associated APase activities were only observed in bean hairy roots with overexpressing *PvPAP1* and *PvPAP3* (Figure 5B and C), suggesting that PvPAP1 and PvPAP3 might participate in extracellular dNTP utilization. Consistent with this hypothesis, the highest dry weight and total P content were observed in *OX-PvPAP1* and *OX-PvPAP3* transgenic hairy roots with 250 µM dNTP application (Figure 6). Furthermore, significantly increased fresh weight and total P content in both *PvPAP3*-overexpression transgenic Arabidopsis lines were also observed when dNTPs were supplied as the sole P source in the media (Figure 7). All results together suggest that PvPAP1 and PvPAP3 might be involved in augmentation of acquisition of Pi from extracellular dNTPs in organic matter surrounding plant roots. Since constitutive and Pi-starvation induced expression patterns were separately found for *PvPAP1* and *PvPAP3* in roots of two bean genotypes (Figure 4), it was suggested that PvPAP1 might function under normal conditions, while PvPAP3 might be regulated to function under Pi-starvation conditions. It has been suggested that PvPAP1 and PvPAP2 might be involved in seed germination of bean because transcripts of *PvPAP1* and *PvPAP2* were separately increased in embryonic axis and cotyledons during seed germination [37], [38]. Therefore, it might be possible that PvPAP1 might exhibit multiple functions in bean except to participate in extracellular dNTP utilization.

Although PvPAP5 exhibited high homology with PvPAP3, and similar subcellular localization with PvPAP1 (Figure 1 and 2), dry weight and total P content of OX-PvPAP5 did not exhibit significant difference from those in CK with 250 µM dNTP application (Figure 6). It might be mainly because no significant increase of root associated APase activities was observed in OX-PvPAP5 hairy roots compared to CK (Figure 5). Another possible reason is that PvPAP5 might exhibit limited activities against dNTPs, but have other specific substrates in plants. Therefore, PvPAP5 could not hydrolyze Pi from dNTPs for plant utilization. It has been documented that different PAPs have various specific substrates [9], [13], [20]. For example, AtPAP15 exhibited high activity against phosphoenolpyruvate, but showed neglectible activities against monophosphates (e.g., adenosine monophosphate) [20].

In the present study, internal APase activities were increased at 8 d after Pi starvation in both the roots and leaves of G19833 and DOR364 (Figure 3). Furthermore, along with the increase of APase activities, expression levels of the three small *PvPAPs* were strongly increased by Pi starvation in both roots and leaves of DOR364 and G19833 except that no response of *PvPAP4* to Pi starvation was observed in the roots of DOR364 (Figure 4), suggesting that the increased transcripts of *PvPAPs* might result in accumulation of corresponding proteins and increase of internal APase activities in plants under low P conditions. It was further supported by our results, in which overexpressing *PvPAP1* and *PvPAP3-5* led to significant increase of internal APase activities in transgenic bean hairy roots (Figure 5). Additionally, other recent work supported that the increases of APase activities in leaves and roots of G19833 and DOR364 are partially caused by a newly synthesized APase isoform, which is encoded by *PvPAP3* [27], [39]. Interestingly, APase activities in the leaves of DOR364 were higher than those in G19833 throughout the duration of P deficiency (Figure 3). This appears to be mainly caused by one major APase isoform existing in the leaves of DOR364, but missing in G19833 [27], [39]. Although the major APase isoform is suggested not to be correlated to superior P efficiency in bean, the gene encoding the major APase isoform and its function remains unclear [39]. Since transcripts of all the tested *PvPAPs* except *PvPAP2* were observed in the leaves of both G19833 and DOR364, we conclude that higher APase activities in the leaves of DOR364 could not be contributed by the accumulated transcripts of the tested *PvPAPs*.

In conclusion, our present results elucidated that PvPAPs in bean varied in protein structure, response to P deficiency and subcellular localization. Among them, transcripts of small *PvPAPs* responded greatly to P efficiency in common bean. PvPAP1 and PvPAP3 appeared to be involved in enhancing Pi mobilization from extracellular dNTPs for plant utilization.

## Materials and Methods

### Data Base Search, Cloning of *PvPAP5* Full Length cDNA and Phylogenetic Analysis

The reported nucleotide sequences of genes encoding PvPAP proteins (accession number: BAD05166, CAA04644, AC025293 and AAF60317) of common bean were downloaded from the NCBI website (http://www.ncbi.nlm.nih.gov/i/). Using these nucleotide sequences as query sequences, Blastn analysis was conducted at the Dana Farber Cancer Institute (DFCI) website (http://compbio.dfci.harvard.edu/tgi/). The analysis results led to retrieval of one new EST (FD786924), predicted to be the fragment of a new gene. Based on the EST sequence, the full length cDNA of *PvPAP5* was cloned from the cDNA library of G19833 subjected to Pi starvation using the method described previously [25]. Briefly, One pair of primers were designed for PCR amplification of *PvPvPAP5* (Forward, 5-GACTTAGAATTGGCACTGAA-3, and reverse, 5- CAGCTTCTCTACAAGTTCTTG -3). PCR amplified cDNA fragments of *PvPAP5* were cloned into a pGEM-T vector and sequenced. The *PvPAP5* sequence was deposited in the NCBI database (GQ891043). In total, five PvPAPs were used for phylogenetic tree analysis, including PvPAP1 (BAD05166), PvAP2 (CAA04644), PvPAP3 (AC025293), PvPAP4 (AAF60317) and PvPAP5 (ADK56125). Multiple sequence alignment was conducted using ClustalW 1.8. A phylogenetic tree was established using the neighbor-joining method of the MEGA 4.1 program.

### Plant Materials and Growth Conditions

Two common bean genotypes contrasting in P efficiency, G19833 and DOR364, were employed. G19833 has been characterized as a P-efficient genotype with better adaptation to low P soils, and DOR364 is a P-inefficient genotype [39], [40]. For time course experiments, seeds of G19833 and DOR364 were surface sterilized for 1 min in 3% (v/v) saturated H_2_O_2_ solution, then germinated in dark on germination papers with modified nutrient solution [39], [40] containing (in µM) 3000 KNO_3_, 2000 Ca(NO_3_)_2_, 500 KH_2_PO_4_, 250 MgSO_4_, 25 MgCl_2_, 12.5 H_3_BO_3_, 1 MnSO_4_, 1 ZnSO_4_, 0.25 CuSO_4_, 0.25 (NH_4_)_6_Mo_7_O_24_, and 25 Fe-Na-EDTA. Seven days after germination, uniform seedlings were hydroponically grown in the same nutrient solution as described above. After 7 d, seedlings were subjected to Pi starvation by supplying 5 µM KH_2_PO_4_. Leaves and roots were harvested separately at 0, 4 and 8 d after the initiation of Pi starvation for further analysis. In all experiments, four replicates were applied.

### Subcellular Localization of *PvPAPs-GFP* Fusion Protein

The coding regions of *PvPAPs* were amplified, digested with *Xba* I, *Kpn* I or *BamH* I and then cloned into the binary EGFP vectors [41]. The plasmids of all *35S:PvPAPs-GFP* constructs and *35S:GFP* empty vector were transformed into onion epidermal cells by the method described before [27]. The transformed epidermal cells were cultured on Murashige and Skoog (MS) medium for 16 h before GFP signal observation. Plasmolysis was conducted by incubating the transformed cells in 30% sucrose. The cell wall was distinguished by propidium iodide (PI) staining. Cells were observed by using a fluorescence microscope (LEICA DM5000B, Germany) and photographed using a LEICA DFC 480 camera (LEICA, Germany).

### APase Activity Assays

Internal APase activities were determined by measuring the release of nitrophenol from p-nitrophenylphosphate (p-NPP) in 45 mM Na-acetate buffer (pH 5.0) as previously described [39]. Briefly, protein extracts were mixed with 2 mL Na-acetate buffer (45 mM, pH 5.0) containing 1 mM p-NPP and incubated at 37°C for 15 min. The reaction was stopped by adding 1 mL of NaOH (1 M) and the absorbance was measured at 405 nm. Protein concentration was determined by the coomassie blue method [42]. For measuring root associated APase activities, transgenic bean hairy root tips (about 3 cm in length) were incubated in 45 mM Na-acetate buffer (pH 5.0) containing 2 mM p-nitrophenylphosphate (p-NPP). After 30 min, the reaction was stopped by adding 1 mL of NaOH (1 M) and the absorbance was measured at 405 nm. Fresh weight of root tips was determined. Four biological replicates were included for each transgenic line.

### Quantitative Real-time PCR (qPCR) Analysis

Total RNA was extracted from each sample using Trizol reagent according to the manual (Invitrogen Inc., USA). After treated with *DNase* I (Invitrogen Inc, USA), 1 µg RNA was used for first strand cDNA synthesis by following the reverse transcription system technical bulletin (Promega Cor, USA). The qPCR analysis was performed using SYBR Green monitored qPCR (Toyobo, Inc., Japan) and a Rotor-Gene 3000 qPCR system (Corbett Research, Australia). The primer pairs used for qPCR analysis were shown in Table S1. All of the gene expression analysis had three biological replications. Expression was calculated from relative expression levels of *PvPAPs* to expression levels of the reference gene *EF-1α* (PvTC3216 from the Dana-Farber Cancer Institute Computational Biology and Functional Genomics Laboratory).

### Development of Transgenic Bean Hairy Roots Overexpressing PvPAPs

For overexpression of *PvPAP1*, *PvPAP3*, *PvPAP4* and *PvPAP5* driven by the 35S promoter, the coding regions of *PvPAP1*, *PvPAP3*, *PvPAP4* and *PvPAP5* were separately amplified with primers (Shown in Table S1). The amplified fragments were digested with *Hind* III and *Mlu* I for *PvPAP1* and *PvPAP3*, *BamH* I and *Mlu* I for *PvPAP4* and *PvPAP5*, respectively. These fragments were subsequently cloned into the binary vector. Common bean hairy roots were produced as described before [27]. Briefly, the seeds of DOR364 were surface-sterilized and were germinated on 1/2 MS medium in dark for 35 to 48 h. The abaxial of cotyledons were wounded with a scalpel previously dipped into the overnight cultures of the *A. rhizogenes* strain (K599) containing *35S:PvPAP1*, *35S:PvPAP3*, *35S:PvPAP4*, *35S:PvPAP5* or the empty binary vector as control. The wounded cotyledons were incubated with abaxial side up on filter paper pre-moistened in sterilized water for 3 d. Cotyledons were then transferred to a solid MS medium containing 500 µg.mL^−1^ carbenicillin disodium and 20 µg.mL^−1^ hygromycin to established hairy roots. After assays of qPCR analysis, one independent transgenic line as one biological replicate for each *PvPAP* and one empty vector control (CK), were separately selected and regenerated for further analysis. In the dNTP utilization experiments, 0, 50 and 250 µM dNTPs were used as the sole external P source, dry weight of the hairy roots from control and the overexpressors with *PvPAP1*, *PvPAP3*, *PvPAP4* and *PvPAP5* was separately analyzed at 7 d after growth. Phosphorus content was analyzed colorimeterically after ash digestion [43]. Each treatment for each OX-PvPAP and CK had five biological replicates.

### Transgenic Arabidopsis Overexpressing *PvPAP3*

The overexpression construct of *PvPAP3* was transformed into *Agrobacterium tumefaciens* strain Gv3101 using the freeze-thaw method, which was subsequently introduced into Arabidopsis plants as previously described [44] to obtain *PvPAP3*-overexpressing plants. Homozygous T3 transgenic seeds of two lines (OX-1 and OX-2) were used for further studies. Ecotype Columbia (Col-0, as wild-type) and *PvPAP3*-overexpression seeds were surface sterilized, and then germinated on Petri dishes containing modified MS solid medium. After 7 d, uniform seedlings were selected and transplanted to new dishes containing the fresh MS solid medium without KH_2_PO_4_ supplication. In the dNTP utilization experiments, 0, 50 and 250 µM dNTPs were separately used as the sole external P source. Seedlings were harvested for further analysis after 7 d of P treatments. Each treatment had four biological replicates, and each replicates had five seedlings.

All the data were analyzed by ANOVA using SAS (Statistical Analysis Systems Institute, version 6.12).

## Supporting Information

Figure S1 **Alignment of PvPAP proteins in common bean.** A) High molecular mass PvPAP1 and PvPAP2; B) Low molecular mass PvPAP3, PvPAP4 and PvPAP5. Five conserved motifs of PvPAPs are boxed. Seven bold letters representing metal-coordinated residues are highlighted. Well conserved residues are indicated by star symbols.
(TIF)

Table S1 **Primers were used for qRT-PCR.**
(DOC)
