# Supplementary figures and images for: Comparative Analysis of PvPAP Gene Family and Their Functions in Response to Phosphorus Deficiency in Common Bean
Source: PLoS One. 2012 May 25;7(5):e38106. doi: 10.1371/journal.pone.0038106 (PMC3360649; doi:10.1371/journal.pone.0038106)

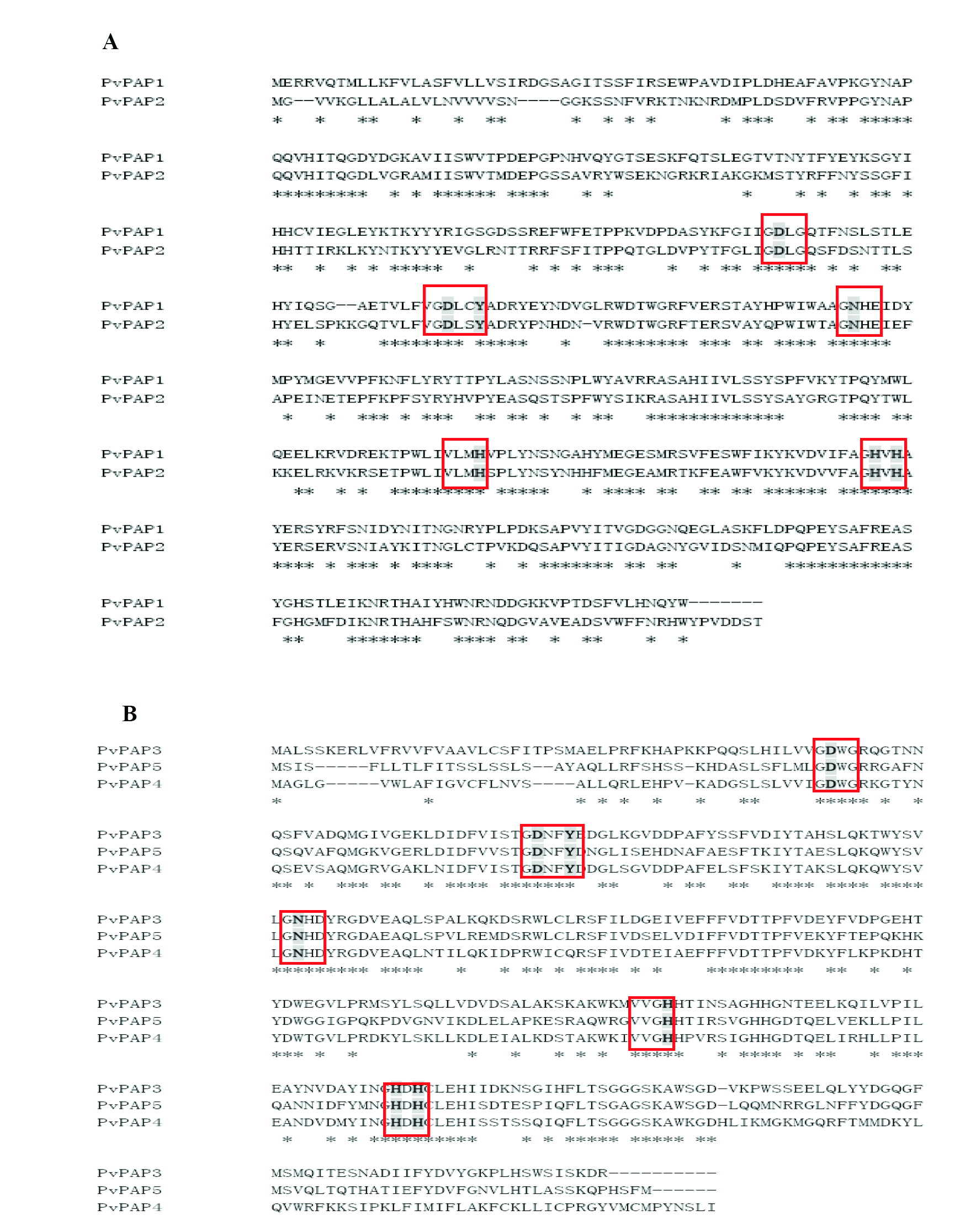

Supplement: Figure S1 — Alignment of PvPAP proteins in common bean. A) High molecular mass PvPAP1 and PvPAP2; B) Low molecular mass PvPAP3, PvPAP4 and PvPAP5. Five conserved motifs of PvPAPs are boxed. Seven bold letters representing metal-coordinated residues are highlighted. Well conserved residues are indicated by star symbols. (TIF) [file pone.0038106.s001.tif]
