# Supplementary material for: Comparative Analysis of PvPAP Gene Family and Their Functions in Response to Phosphorus Deficiency in Common Bean
Source: PLoS One. 2012 May 25;7(5):e38106. doi: 10.1371/journal.pone.0038106 (PMC3360649; doi:10.1371/journal.pone.0038106)
Supplement: Table S1 — Primers were used for qRT-PCR. (DOC) [file pone.0038106.s002.doc]

| Gene | GeneBank Accession number | Forward Primer (5' - 3') | Reverse Primer (5' - 3') |
| --- | --- | --- | --- |
| *PvPAP1* | BAD05166 | GCTTCCTTTGTCCTCTTGGT | TGTAACTGTGCCTTCTAAACTGG |
| *PvPAP2* | CAA04644 | GAGTAATGGAGGCAAAAGCAGCAAC | CATTGCCCTCCCCACAAGGTCAC |
| *PvPAP3* | AC025293 | GAACGCTTGGTATTCCGTGT | TGTCTTCCCCAATCTCCAAC |
| *PvPAP4* | AAF60317 | GGTGTATGGTTGGCTTTCATTGGTG | GCAGAAACTTCAGATTGGTTGTAGG |
| *PvPAP5* | ADK56125 | GGAGGGGAGTTGTTGGTCAC | ACCCTGCTCCGCTAGTCAAG |
| *EF-1α* | TC3216* | TGAACCACCCTGGTCAGATT | TCCAGCATCACCATTCTTCA |

*The number of *EF-1α* was from DFCI Computational Biology and Functional Genomics Laboratory

Supplemental table2 Primers were used for constructing overexpression vector

| Gene | Forward Primer (5' - 3') | Reverse Primer (5' - 3') |
| --- | --- | --- |
| *PvPAP1* |  |  |
| *PvPAP3* | TCAGAAGCTTGGCATGGCGTTATC | CTGACGCGTGATTCTTTACCGGTCTTT |
| *PvPAP4* | GGATCCTATGGCTGGTTTGG | ACGCGTTTAAATAAGGGAGTTATAT |
| *PvPAP5* | GGATCCTATGTCCATCTCCTTT | ACGCGTTTACATGAAAGAATGTG |
